# Facilitators and Barriers to Passing Local Policies That Prohibit the Sale of Flavored Tobacco Products: Qualitative Analysis of Strategies Implemented by 36 Communities in California, 2017–2021

**DOI:** 10.5888/pcd21.230283

**Published:** 2024-05-30

**Authors:** Sarah Hellesen, Sue Haun, Melanie S. Dove

**Affiliations:** 1 Public Health Sciences, School of Medicine, University of California, Davis, California

## Abstract

To reduce youth access to tobacco products, the California Tobacco Prevention Program funded local tobacco prevention programs from July 2017 through December 2021 to address its Communities of Excellence Indicator 3.2.9: “the number of jurisdictions with a policy eliminating or restricting the sale and/or distribution of any mentholated cigarettes and other flavored tobacco products, and paraphernalia.” We examined the strategies by which community coalitions attempted to limit the number of stores selling flavored tobacco across California. Thirty-six final evaluation reports (FERs) were used for our analysis. We examined certain elements or factors as primary areas of interest because of their apparent link to successful outcomes in analyses of FERs in the past. Over half (19 of 36) of FERs reported successfully passing at least 1 policy to regulate the sale of flavored tobacco products. Urban communities passed more policies (16 of 18) compared with rural communities (3 of 18). Successful campaigns tended to involve youth, demonstrate illegal sales to minors and public support for a ban, and identify a champion. Barriers included the COVID-19 pandemic, California wildfires, staffing shortages, and conservative political climates. This evaluation offers insights into the successes and challenges faced by local coalitions seeking policy changes for tobacco use prevention, which can be different for urban and rural communities. The evaluation also indicates the necessity of adopting flexible tactical plans for overcoming environmental factors that affected intervention and evaluation activities.

**Summary**

**What is already known on this topic?**

Passing policies that prohibit the sale of flavored and menthol tobacco products is associated with a decrease in youth and young adult tobacco use.

**What is added by this report?**

The COVID-19 pandemic, California wildfires, staffing shortages, and conservative political climates represented significant barriers to policy adoption. Successful campaigns tended to demonstrate illegal sales to minors and public support for a ban. Urban communities passed more policies restricting flavored tobacco sales than rural communities did.

**What are the implications for public health practice?**

Passing future restrictions on tobacco sales will require tailoring interventions to communities’ political climates and adapting work plans to be more flexible in the event of future emergencies and interruptions.

## Introduction

Commercial tobacco use remains the leading cause of preventable death and disease in the United States (1). Preventing initiation and ongoing use of tobacco products by young people is critical, as most adults who use tobacco begin before the age of 18 years (2). Young tobacco users overwhelmingly use flavored tobacco products, including products with menthol (3), which improves the taste and reduces the harshness of the tobacco product, making them more appealing to new users (2).

The California Department of Public Health, Tobacco Prevention Program (CTPP) aims to change tobacco-related social norms by creating an environment where “tobacco becomes less desirable, less acceptable, and less accessible” (4). CTPP funds tobacco use prevention programs in all 58 counties as well as 3 cities in California to focus on 1 or more policy objectives that fall under its 4 priority areas: limit tobacco-promoting influences, reduce exposure to secondhand smoke, reduce the availability of tobacco, and promote tobacco use cessation (4).

CTPP-funded objectives require programs to follow approved scopes of work and submit required intervention and evaluation deliverables every funding cycle that align with its Communities of Excellence Indicators (4,5). These deliverables include collecting data (eg, public opinion, key informant, observation data), providing educational materials and resources relevant to tobacco use prevention to their community coalitions, and submitting evaluation reports on their community’s progress (4). The role of a community coalition in tobacco use prevention is to provide a strong voice for policy change on behalf of those who live in the target jurisdictions. Coalition members educate, make presentations to, and communicate with policymakers to campaign for policy change.

From July 2017 through December 2021, as part of its effort to reduce youth access to tobacco products, CTPP funded 36 local public health programs to address Communities of Excellence Indicator 3.2.9: “the number of jurisdictions with a policy eliminating or restricting the sale and/or distribution of any mentholated cigarettes and other flavored tobacco products, and paraphernalia” (5).

## Purpose and Objectives

The primary objective of our evaluation was to determine the facilitators and barriers to successful adoption of local policies that prohibit the sale of flavored tobacco products. At the conclusion of the 2017–2021 funding cycle, 36 local public health programs produced Final Evaluation Reports (FERs) describing their coalition’s experience and reporting whether they succeeded in meeting their objectives. We report on the common factors that were found to be instrumental in their campaigns or that served as obstacles.

## Intervention Approach

The evaluation team’s analysis examined certain elements or factors as primary areas of interest due to their apparent link to successful outcomes in past analyses of FERs (6,7). These are the involvement of youth, use of media for education and advocacy, involvement of policymakers and law enforcement, and gathering data through youth tobacco purchase survey results, public opinion surveys, and tobacco retail store observations.

## Evaluation Approach

The data used in the qualitative analysis were drawn exclusively from the 36 FERs as they were submitted at the end of the funding cycle. FERs followed a standard format comprising an abstract, background, evaluation methods and design, implementation and results, and conclusions and recommendations. The evaluation team analyzed the content of these FERs; codes were used to identify common categories, themes, and relationships (8).

The evaluation team also analyzed the demographic data and tobacco control characteristics for each community to determine if patterns were present (Table 1). The total population, the land area in square miles, and the rural/urban status were obtained from the 2017–2021 American Community Survey, the 2010 Census, and the Rural Initiatives Strengthening Equity (9), respectively. The 2019 overall tobacco control grade (A–F) came from the American Lung Association (10). The percentage of adults who smoked cigarettes (2016–2018 California Health Interview Survey) was also included (Table 1).

**Table 1:** Demographic and Tobacco Control Characteristics for Each Community Prohibiting the Sale of Flavored Tobacco Products, California, 2017–2021 (N = 36)

County or city^a^	Population^b^	Land area, square miles^c^	Tobacco control grade^d^	Adult smokers, %^e^
**Rural**				
Alpine County	NA	738.33	D	NA
Calaveras County	45,349	1,020.01	F	13.0
Colusa County	21,780	1,150.73	F	15.1
Inyo County	18,804	10,180.88	F	13.0
Lassen County	32,949	4,541.18	F	16.1
Madera County	156,304	2,137.07	F	13.0
Mariposa County	17,225	1,448.82	D	NA
Mendocino County	91,534	3,506.34	D	15.1
Merced County	279,150	1,934.97	F	11.0
Plumas County	19,631	2,553.04	F	16.1
San Joaquin County	771,406	1,391.32	F	11.8
Sierra County	NA	953.21	F	16.1
Sutter County	99,080	602.41	D	14.1
Tehama County	65,345	2,949.71	F	15.1
Yuba County	80,404	631.84	F	17.5
**Butte County**	217,884	1,636.46	D	17.0
**Modoc County**	8,723	3,917.77	D	16.1
**Shasta County**	181,935	3,775.40	D	20.1
**Urban**				
Fresno County	1,003,150	5,957.99	F	10.1
San Benito County	63,329	1,388.71	F	14.2
**Alameda County**	1,673,133	739.02	B	8.2
**Berkeley City**	119,607	10.47	A	NA
**Contra Costa County**	1,161,643	715.94	C	9.4
**Long Beach City**	466,565	50.29	C	NA
**Los Angeles County**	10,019,635	4,057.88	D	8.6
**Marin County**	262,387	520.31	B	9.8
**Monterey County**	438,953	3,280.60	D	8.3
**Napa County**	138,795	748.36	F	8.2
**Sacramento County**	1,571,767	964.64	C	8.8
**San Bernardino County**	2,171,071	20,056.94	F	12.3
**San Luis Obispo County**	282,771	3,298.57	C	8.1
**Santa Clara County**	1,932,022	1,290.10	C	5.1
**Santa Cruz County**	272,138	445.17	B	10.2
**Sonoma County**	492,498	1,575.85	B	9.3
**Ventura County**	845,255	1,843.13	D	8.2
**Yolo County**	216,703	631.84	C	5.4

Abbreviation: NA, not available because of small population size or because the data were not available at the city level.

a County names in bold indicate that the county passed a ban on flavored tobacco products. Rural or urban status was obtained from Rural Initiatives Strengthening Equity (9).

b 2017–2021 American Community Survey.

c 2010 US Census.

d 2019 American Lung Association Tobacco Control Grade.

e 2017–2019 California Health Interview Survey — percentage of adults (≥18 years) who currently smoke.

## Results

Of the 36 FERs that reported on policies to prohibit the sale of flavored tobacco, 19 (53%) stated that their communities were successful in meeting their stated objectives. Urban communities passed more policies (16 of 18) than rural communities (3 of 18).

### Facilitators of policy change

Key facilitators of policy change included the involvement of youth, identifying policy champions, involving a community coalition, sharing data to demonstrate need and support for a policy, and using precedents (Table 2).

**Table 2:** Facilitators to Passing a Flavored Tobacco Sales Restriction to Prohibit the Sale of Flavored Tobacco Products, California, 2017–2021 (N = 36)^a^

County or city^a^	Facilitators			
	Involved youth	Recruited a champion	Involved or broadened the county coalition	Shared data^b^
**Rural**				
Alpine County	Y	NA	NA	Y
Calaveras County	Y	Y	Y	Y
Colusa County	Y	NA	Y	Y
Inyo County	Y	NA	NA	Y
Lassen County	Y	NA	Y	NA
Madera County	Y	Y	NA	Y
Mariposa County	Y	NA	Y	NA
Mendocino County	Y	NA	NA	NA
Merced County	Y	NA	Y	NA
Plumas County	Y	Y	Y	Y
San Joaquin County	Y	NA	Y	Y
Sierra County	N	NA	NA	NA
Sutter County	Y	NA	Y	NA
Tehama County	Y	Y	Y	Y
Yuba County	Y	NA	Y	Y
**Butte County**	Y	Y	Y	Y
**Modoc County**	N	Y	Y	Y
**Shasta County**	Y	NA	Y	Y
**Urban**				
Fresno County	Y	NA	NA	Y
San Benito County	Y	Y	Y	NA
**Alameda County**	Y	Y	Y	Y
**Berkeley City**	Y	NA	Y	Y
**Contra Costa County**	Y	NA	Y	NA
**Long Beach City**	Y	NA	NA	NA
**Los Angeles County**	Y	Y	Y	Y
**Marin County**	Y	NA	Y	Y
**Monterey County**	Y	Y	Y	Y
**Napa County**	Y	NA	Y	Y
**Sacramento County**	Y	Y	Y	NA
**San Bernardino County**	Y	NA	Y	Y
**San Luis Obispo County**	Y	Y	Y	Y
**Santa Clara County**	Y	NA	Y	Y
**Santa Cruz County**	Y	Y	Y	NA
**Sonoma County**	Y	Y	Y	Y
**Ventura County**	Y	Y	Y	Y
**Yolo County**	Y	Y	NA	Y

Abbreviation: N, facilitator was not used; NA, facilitator was not reported in a final evaluation report or was not used by the county or city; Y, facilitator was used.

a County names in bold indicate that the county passed a ban on flavored tobacco products. Rural or urban status was obtained from Rural Initiatives Strengthening Equity (9).

b Shared data in community presentations, fact sheets, and educational outreach packets; with policymakers and coalition members; and through media press releases or social media (see Appendix for data points).

#### Involvement of youth

Almost all (34 of 36) FERs reported engaging youth. For example, 1 successful coalition recruited, trained, and used 87 youth volunteers to conduct a house-to-house door hanger campaign, create public service announcements, and develop an op-ed column or a letter to the editor.

Youth were primarily involved in conducting Young Adult Tobacco Purchase Surveys in their communities. These surveys were used to assess and document the rate of illegal sales to underage persons. Of the 36 programs, 18 included a Young Adult Tobacco Purchase Survey as part of their 2017–2021 scope of work, and 15 reported their results in the FER.

Most communities were able to document the problem of the rate of illegal sales to minors. Preintervention illegal sales ranged from 0% to 57%. The ability to demonstrate that illegal sales were a problem in the local community was reported as a facilitator of policy change. Coalitions that were unable to make the case that illegal sales were a problem in the community were less successful in their policy change efforts. One rural county’s FER reported an illegal sales rate of 6% (2 of 34 tobacco retailers). Another rural county had only 3 tobacco retailers countywide. In each case, policymakers did not believe the data supported the case for policy change.

#### Identifying policy champions

The role of a champion is to advocate for the adoption of a policy from within the decision-making body. Several FERs mentioned having strong champions from the city council “who assisted in spreading knowledge about the potential policy to community members and their fellow council members.” Because it is possible to lose a policy champion if priorities shift or crises arise, one FER noted that “it is absolutely critical to have more than one council member championing the issue.”

#### Involving a community coalition

As community coalitions led policy change efforts, most FERs mentioned the importance of adult and youth coalitions. One FER noted that “building strong community support and collaborative partnerships was critical” to the passage of policies in 3 jurisdictions. Another stated, “The combination of champions, allies, and volunteers snowballed into momentum that was also powered by media advertisements and press releases.”

### Demonstrating need and support for a policy

Seeing public support for reducing youth access and adopting flavored tobacco product bans is important to policymakers. Several FERs reported their communities conducted public opinion polls, gathered petition signatures, and conducted letter-writing campaigns. For example, one county’s youth advocates collected endorsements of support. The results of these efforts were included in information kits, communicated during presentations, or submitted to local media for release.

Twenty-five of the programs reported the results of the public opinion surveys. The percentage of the surveyed public that was in support of the ban varied from 47% to 90%. Some programs used the results in fact sheets, presentations, and community or policymaker education. However, not all programs were able to do so, and in the jurisdictions that did not pass a policy, it appears that the results were not shared with the community. For example, in one FER, although 72% of the residents surveyed were in support of a flavored tobacco products ban, the results were not used because of redirection of staff to COVID-19 pandemic–related duties, unresponsiveness of policymakers, and later turnover in staff.

#### Using precedents

Coalitions found it beneficial to build on existing laws and precedents. Some FERs reported that lawmakers were influenced by policy discussions and policies being passed in neighboring counties or jurisdictions. For example, 1 FER mentioned that the community “benefitted from efforts from other local cities, which was referenced not only by city council members but from community comments, as well.” Providing examples from similar counties when educating the community and policymakers was noted in some FERs because policymakers want to see examples of success.

### Barriers to policy adoption

The communities faced barriers to policy adoption that delayed intervention activities or prevented them altogether. These barriers included long adoption timelines, environmental factors, and the conservative political climates in some jurisdictions.

#### Policy change takes time

The length of time to get a proposed policy introduced, let alone accepted and implemented, was one challenge. This can be compounded by other barriers that delay the process. For example, some FERs reported the resignation of staff or positions that were not filled for multiple years. Programs that faced staffing shortages or high staff turnover, lost their policy champion, or were unable to keep their community coalition engaged long-term had difficulty maintaining the momentum necessary to address their Communities of Excellence Indicator.

#### Environmental factors

Events outside of the coalitions’ control can also affect progress toward passing flavored tobacco restrictions. Almost every FER reported that the COVID-19 pandemic interrupted their work; for some, it was a temporary interruption as they were ultimately successful in passing policies, but other communities had not yet recovered at the end of 2021. Policymakers focused on other priorities because of the pandemic, and some coalitions found it difficult to engage schools and parents in campaign efforts. It was also difficult to get the attention of the media. In addition, post-policy adoption education, enforcement, and evaluation activities were delayed or not conducted because of the pandemic, which would have provided valuable information about the level of compliance with flavored tobacco product bans.

Other factors that hindered progress were wildfires and extreme weather. The California wildfires that occurred during 2017–2021 (eg, The Tamarack Fires, the Glass Fires, and the Beckwourth Complex Fires) caused evacuations that delayed intervention and evaluation activities, making it difficult to build momentum. As extreme weather becomes the norm in California (11), local programs and coalitions may need to be more flexible in their approaches to community engagement and data collection.

#### Conservative political climate

FERs from rural counties reported that their policymakers tended to be more conservative politically than urban policymakers. In such jurisdictions, policymakers were hesitant to support initiatives that were perceived as antibusiness or that negatively affected local businesses and had “strong resistance to governmental interference in an individual’s perceived rights and freedoms,” as one FER noted. During this funding period, only 3 of 18 rural communities passed flavored tobacco bans, compared with 16 of 18 urban communities. Because the challenges faced in pursuit of policy change may be very different for urban and rural counties, coalitions pursuing tobacco use prevention will need to collect evaluation data to ascertain the readiness of their community to pass tobacco prevention policies and tailor their intervention activities appropriately.

## Implications for Public Health

Over half of the communities that reportedly attempted to pass policies prohibiting the sale of flavored tobacco were successful. Many FERs reported that lawmakers were influenced by the policy changes occurring in neighboring jurisdictions (12). The importance of understanding local political climates and identifying champions among key parties in the community to push for local policy change is consistent with existing studies on policy change (7,13).

Youth engagement is a critical part of comprehensive tobacco control efforts, because preventing tobacco use initiation among young people is key to ending the tobacco epidemic. These young people help communicate the impact of tobacco on their communities, implement tobacco control strategies, and shift social norms (14).

As reported in the FERs, coalitions in rural areas not only had resistance from more conservative policymakers but also faced unique challenges in completing intervention activities that urban jurisdictions may not face. FERs from rural counties noted that media was limited when a county did not have any major news outlets. In some cases, these coalitions turned to regional outlets or streaming services; media coverage was achieved only because it was purchased.

Conducting data collection to demonstrate a local problem or public support for a policy is more difficult in rural regions as well, both due to logistics and small sample sizes. For rural counties, the geographic distance between communities can be significant. One rural county has a total of 3 tobacco retailers. Another rural county reported an illegal sales rate of 6% (2 of 34 tobacco retailers), which did not make the case for policy change.

This evaluation also indicates the necessity of adopting flexible tactical plans for overcoming environmental factors that influenced intervention and evaluation activities. Wildfires that devastated multiple regions of California during 2017 through 2021, as well as the COVID-19 pandemic, affected efforts to educate the community and decision-makers. These environmental factors also affected the ability to collect the necessary data to demonstrate the need and public support for policy change. These difficulties are indicative of the changing landscape of public health work and highlight the importance of adapting work plans to be more flexible in the event of future emergencies and interruptions.

## Appendix

**Table:** Table. Data Included in Final Evaluation Reports Describing Efforts to Prohibit the Sale of Flavored Tobacco Products, California, 2017–2021 (N = 36)^a^

County or city^b^	Stores that sold tobacco to underage youth, %^c^	Flavored tobacco product availability in stores, %^c^	Public support for a flavor ban, %^c^
**Rural**			
Alpine County	0	100	50
Calaveras County	45	94	57
Colusa County	6, 17	86	72
Inyo County	NR	NR	NR
Lassen County	NR	100, 80	58
Madera County	NR	93	NR
Mariposa County	NR	NR	NR
Mendocino County	NR	NR	NR
Merced County	10, 19	NR	NR
Plumas County	24, 30	80	66
San Joaquin County	18	NR	78
Sierra County	NR	100	47
Sutter County	NR	95, 91	69
Tehama County	NR	98	71
Yuba County	36.6	NR	51
**Butte County**	20, 38	96, 90	66
**Modoc County**	NR	100	83
**Shasta County**	NR	84	62
**Urban**			
Fresno County	NR	100, 97	63
San Benito County	NR	NR	NR
**Alameda County**	NR	94, 100	80
**Berkeley City**	NR	83	67
**Contra Costa County**	NR	NR	NR
**Long Beach City**	NR	NR	NR
**Los Angeles County**	6–48	NR	52–88
**Marin County**	NR	NR	80
**Monterey County**	13–57	90, 82	83
**Napa County**	NR	84, 69	89
**Sacramento County**	NR	89	NR
**San Bernardino County**	20–40	80	77
**San Luis Obispo County**	7–21	80	53
**Santa Clara County**	23	91	68
**Santa Cruz County**	NR	NR	NR
**Sonoma County**	17.1	87, 80	90
**Ventura County**	23–30	NR	NR
**Yolo County**	NR	73	74

Abbreviation: NR, not reported.

^a^ Multiple numbers in a cell represent percentages in multiple jurisdictions in which the programs collected data; eg, Colusa County reported store percentages in 2 jurisdictions, and Los Angeles County reported a range of percentages across each of its jurisdictions.

^b^ County names in bold indicate that the county passed a ban on flavored tobacco products. Rural or urban status was obtained from Rural Initiatives Strengthening Equity (9).

^c^ Data reported in the final evaluation reports (12).
